# Diagnostic utility of LDH measurement for determining the etiology of modified transudate pleural effusion in cats

**DOI:** 10.3389/fvets.2022.1044192

**Published:** 2022-11-03

**Authors:** Hsu Mon Hla, Vachira Hunprasit, Jedsada Siripoonsup, Anudep Rungsipipat, Araya Radtanakatikanon

**Affiliations:** ^1^The International Graduate Program of Veterinary Science and Technology, Faculty of Veterinary Science, Chulalongkorn University, Bangkok, Thailand; ^2^Department of Veterinary Medicine, Faculty of Veterinary Science, Chulalongkorn University, Bangkok, Thailand; ^3^Department of Pathology, Faculty of Veterinary Science, Chulalongkorn University, Bangkok, Thailand; ^4^Center of Excellence for Companion Animal Cancer, Faculty of Veterinary Science, Chulalongkorn University, Bangkok, Thailand

**Keywords:** modified transudate, lactate dehydrogenase, pleural effusion, cat, etiology

## Abstract

Fluid analysis is an initial approach for determining the underlying causes of body cavity effusions. Modified transudate is commonly diagnosed in pleural effusion in cats, however, it provides limited diagnostic information. Aims of this study were to investigate common etiologies causing different pleural fluid types and to evaluate the usefulness of lactate dehydrogenase (LDH) for differentiating the etiology in modified transudates in cats. Pleural effusion samples from 122 cats were analyzed and classified into three types: transudate, modified transudate, and exudate. Causes of pleural effusion were classified into four conditions: cardiac disease, neoplasia, feline infectious peritonitis (FIP), and pyothorax. The relationship of underlying etiology and fluid types was described. The LDH levels in pleural fluid and plasma were compared between the causes in the samples classified as modified transudate. The fluid analysis of pleural effusion showed that modified transudate was the most common fluid type (44.2%). Neoplasia was predominantly diagnosed (38.5%) as the etiology of pleural effusion. There was no significant correlation between pleural fluid and plasma LDH level in any type of pleural fluid, suggesting that pleural fluid LDH does not appear to be affected by plasma LDH. The occurrence of modified transudate was not associated to its etiologies, however, the LDH level in modified transudates showed significant differences between etiologic groups. The LDH level in modified transudate was excellent in separating cardiac from non-cardiac diseases with a cut-off value of <535 U/L and separating FIP from non-FIP diseases with a cut-off value of >641 U/L. Based on the current findings, pleural fluid LDH can be a useful adjunctive marker for differentiating some causes of modified transudate pleural effusion and should be added in the routine diagnostic work-up of feline patients with pleural effusions.

## Introduction

Pleural effusion is a common cause of respiratory distress that brings cats to emergency hospitalization. Body fluid analysis helps clinicians to identify underlying mechanisms of fluid accumulation and provide an initial list of differential diagnosis (1). Light's criteria has been used in human medicine to classify body fluids into transudate and exudate based on pathophysiology (2). Excess fluid accumulation due to increased hydrostatic or decreased osmotic pressure is defined as transudative effusion, while exudative effusion is caused by increased vascular permeability as a consequence of inflammation. Light's criteria utilizes total protein (TP) concentration and lactate dehydrogenase (LDH) activity ratio between serum and pleural fluid to discriminate the fluid types because these parameters reflect pleural microvascular permeability and degree of inflammation (3).

In veterinary medicine, body cavity effusion has been classified into transudate, modified transudate, and exudate based on the TP concentration and total number of nucleated cell count (TNCC) (4–6). While the characteristics of exudate and transudate can be explained by their pathophysiology, the association between pathogenesis and fluid characteristics of the modified transudate has not been clearly described. Modified transudate, also known as protein-rich transudate, develops in response to increased hydrostatic pressure or vascular permeability and can also result from a long-standing transudate (7, 8). The mechanism of formation involves many diseases including congestive heart failure, intrathoracic neoplasm, and feline infectious peritonitis (FIP) (9–11). Although modified transudate is commonly found, it has the least specificity to the causes. This makes the pleural fluid classified as modified transudate less diagnostically beneficial to the clinicians.

Lactate dehydrogenase is a cytoplasmic enzyme that catalyzes the pyruvate conversion to lactate during anaerobic glycolysis. Cell types with different energy requirement contain different LDH level which is minimally distributed in the blood. Increased LDH level in the extracellular spaces such as serum, saliva, cerebrospinal fluid, and body cavity effusion is related to its leakage from damaged cells and can be indicative of a pathologic process (12–14). Analysis of LDH in body fluid has suggested a potential diagnostic biomarker in animals. Elevation of total LDH activity and alteration in its isoenzyme fraction are associated with inflammation, cellular injury, and malignancies (15–17).

There have been limited studies on the prevalence of underlying diseases which are causing distinct types of pleural effusion. Determining the etiology is important for proper treatment and a systematic approach is necessary because the differential diagnosis for pleural effusion is diverse. As LDH plays a key role in cellular energy metabolism, the increase of its concentration in a confined space such as the thoracic cavity possibly indicates local tissue pathology in different diseases. The aims of this study were to investigate common etiologies causing different pleural fluid types and to evaluate the usefulness of LDH to differentiate the etiology in modified transudates.

## Materials and methods

### Sample collection and classification of the effusion

This prospective cross-sectional study was conducted between January 2020 and December 2021. The inclusion criteria were cats with pleural effusion, regardless of age, sex, and breed, that visited the Small Animal Hospital, Faculty of Veterinary Science, Chulalongkorn University (VET CU) for the first time (*N* = 122). The pleural fluid samples obtained by ultrasound-guided thoracocentesis were collected in EDTA or plain tubes. If available, plasma samples were collected from the cats on the same day of thoracocentesis as a part of the clinical work-up. Routine body fluid analysis including cytology, TNCC, and TP concentration were performed by a veterinary clinical pathologist within 6 h after the thoracocentesis. The fluid supernatant and plasma biochemistry including triglyceride (in cases suspected of chylothorax), LDH activity, and TP concentration were determined using biochemistry analyzer (BS-800 Mindray; Shenzhen Mindray Bio-Medical Electronics). According to veterinary classification of body cavity fluid, the pleural effusions were divided into three types (5, 6). In this study, each effusion was classified as transudate when the TP was <2.5 g/dl and the TNCC was ≤1,500/μl; modified transudate when the TP was >2.5 g/dl and the TNCC was >1,500 and ≤7,000/μl; and exudate when the TP was >3 g/dl and the TNCC was >7,000/μl.

### Diagnostic criteria of etiology

Diagnosis of the etiology was given based on the assessment of clinical history, physical examination, diagnostic imaging, hemogram, blood chemistry, cytology, and bacterial culture results retrieved from the hospital electronic medical records. Molecular diagnostics of infectious and lymphoproliferative diseases were applied in clinically indicated cases. Feline leukemia virus (FeLV) antigen and feline immunodeficiency virus (FIV) antibody were tested using lateral flow test (Witness FeLV-FIV; Zoetis). Detection of feline coronavirus (FCoV) by RT-PCR in pleural fluids were performed at the Veterinary Diagnostic Laboratory, VET CU using the protocol described elsewhere (11). Clonality testing of neoplastic lymphoid proliferation was performed at the CE-CAC using the previously published approaches (18). The etiology of the pleural effusion was divided into five common conditions by using the following inclusion criteria of each condition.

Cardiac disease was diagnosed based on clinical history with evidence of cardiomyopathy by echocardiography, thoracic radiography, and/or increased N-terminal pro-B-type natriuretic peptide (NT-proBNP) concentration using lateral flow test (SNAP Feline proBNP test; IDEXX).

Neoplastic effusion was diagnosed by a suggestive radiography of the mass in the thoracic cavity and histopathology or cytology of neoplastic cells in the effusion. Diagnosis of malignant epithelial tumor was given to the cases when neoplastic epithelial cells were found with or without mild neutrophilic inflammation. Diagnosis of lymphoma was given to the cases with inconclusive lymphoproliferative lesion when clonality testing was suggestive.

Feline infectious peritonitis was diagnosed by supportive signalment, clinical signs, presence of protein-rich pleural fluid, and positive results of RT-PCR testing for FCoV in the effusions.

Pyothorax was diagnosed when intracytoplasmic bacteria were observed in cytology or a bacterial culture from pleural fluid sample was suggestive.

Chylothorax was diagnosed based on physical characteristics such as milky appearance, predominant presence of small lymphocytes and lipid droplets on cytology, and the triglyceride concentration >100 mg/dl (5).

### Statistical evaluation

The statistical analyses were performed using SPSS Statistics for Windows (Version 28.0. Armonk; IBM). Descriptive analysis and contingency table were used to summarize the relationship between pleural fluid type and underlying etiology. The normality of the data was evaluated by Kolmogorov-Smirnov test. Log transformation of LDH activity was used. The correlation of log (LDH) and TP between pleural fluid and plasma was evaluated using Pearson's correlation coefficient. Data from modified transudate were selected to compare between log (LDH) and etiologies using one-way ANOVA and Tukey's *post-hoc* test. Receiver operating characteristic (ROC) curve analysis was used to plot sensitivity and one-specificity for distinguishing the underlying causes of modified transudate and the optimal cut-off values were selected based on Youden's index (19, 20). The sensitivity and specificity were expressed as percentage and 95% confidence interval (CI). The result was significantly different if the *P*-value <0.05.

## Results

### Fluid type classification and etiology

**Figure 1:**
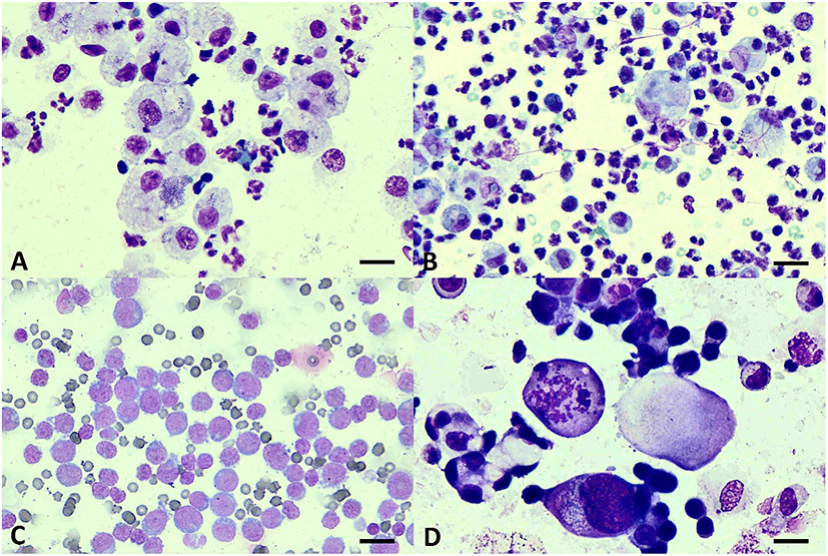
Cytology of pleural effusions. **(A)** Pyogranulomatous effusion from pyothorax showing intracytoplasmic cocci bacteria in vacuolated macrophages and degenerate neutrophils. **(B)** FIP effusion showing non-degenerate neutrophils, macrophages, and few small lymphocytes. **(C)** Monomorphic population of large lymphoblasts suggestive of lymphoma. **(D)** Clusters of epithelial cells showing criteria of malignancy including anisocytosis, pleomorphism, and atypical mitosis suggestive of malignant epithelial tumor. Giemsa staining. Scale bars = 20 μm **(A)**; 30 μm **(B–D)**.

A total of 122 cats with pleural effusions were enrolled in the study. Modified transudate was the most common fluid type (44.2%, 54/122), followed by exudate (42.6%, 52/122) and transudate (13.1%, 16/122). Out of 122 cats with pleural effusion, neoplasia represented the largest group of the patients (38.5%, 47/122), followed by cardiac diseases (18.7%, 24/122), FIP (18%, 22/122), pyothorax (12.3%, 15/122), and chylothorax (1.6%, 2/122). Four cats were diagnosed with more than one etiology (two cats had cardiac disease with neoplasia and two cats had cardiac disease with pyothorax) and eight cats had unknown etiology due to insufficient clinical information. Cytology of pleural fluid diagnosed with pyothorax, FIP, and neoplasia was shown in Figure 1. The most common neoplasm found in this study was lymphoma (82.9%, 39/47) and 64.1% (25/39) of the lymphoma cases were FeLV positive. The other neoplasms found in this study were metastatic mammary gland tumor (12.8%, 6/47) and malignant epithelial tumor suspecting mesothelioma (4.2%, 2/47). Numbers of the cats with a definitive diagnosis (*N* = 108) as neoplasia, cardiac disease, FIP, and pyothorax were displayed with the frequency of the pleural fluid type in a contingency table (Table 1). Transudate effusion was found only in cardiac disease and neoplasia diagnosed as malignant epithelial tumor (data not shown). Modified transudate was found in cats diagnosed with neoplasia, cardiac disease, and FIP. Exudate was not found in cat diagnosed with cardiac disease. Biochemical parameters in pleural fluid and plasma in each etiology were provided in the Supplementary material.

**Table 1:** Contingency table displays the frequencies of pleural fluid type and etiology in cats with definitive diagnosis (*N* = 108).

**Type of fluid**	**Causes**			
	**Neoplasia**	**CD**	**FIP**	**Pyothorax**
Transudate	2	10	0	0
Modified transudate	16	14	17	0
Exudate	29	0	5	15
Total	47	24	22	15

CD, cardiac disease; FIP, feline infectious peritonitis.

### Correlation of LDH level between pleural fluid and plasma

**Figure 2:**
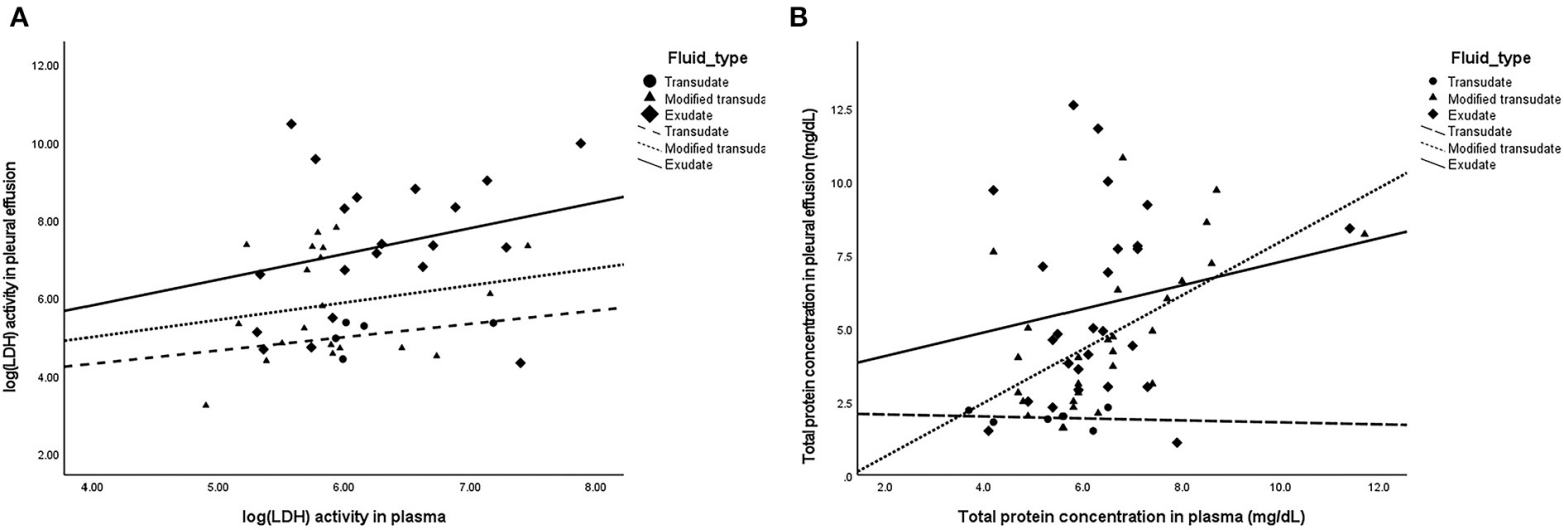
Scatter plots demonstrating the correlation of parameters in pleural fluid and plasma. **(A)** No significant correlation between pleural fluid and plasma LDH levels (Overall: *r* = 0.298; *P* = 0.05) in transudates (*r* = 0.451; *P* = 0.446), modified transudates (*r* = 0.203; *P* = 0.391), and exudates (*r* = 0.269; *P* = 0.252). **(B)** Significant correlation between pleural fluid and plasma TP concentration (Overall: *r* = 0.394; *P* < 0.01) in exudates (*r* = 0.176; *P* = 0.391), modified transudates (*r* = 0.585; *P* < 0.01), and transudates (*r* = −0.129; *P* = 0.808).

The Pearson's correlation illustrated scattered plots of TP concentration and LDH level in pleural fluid and plasma (Figure 2). There was no significant correlation of LDH levels in pleural fluid and plasma (overall *r* = 0.298; *P* = 0.05) in all fluid types. Significant correlation of TP in pleural fluid and plasma was observed (overall *r* = 0.394; *P* < 0.01), particularly in the modified transudate fluid (*r* = 0.585; *P* < 0.01).

### Analysis of LDH values in cats with modified transudates

**Figure 3:**
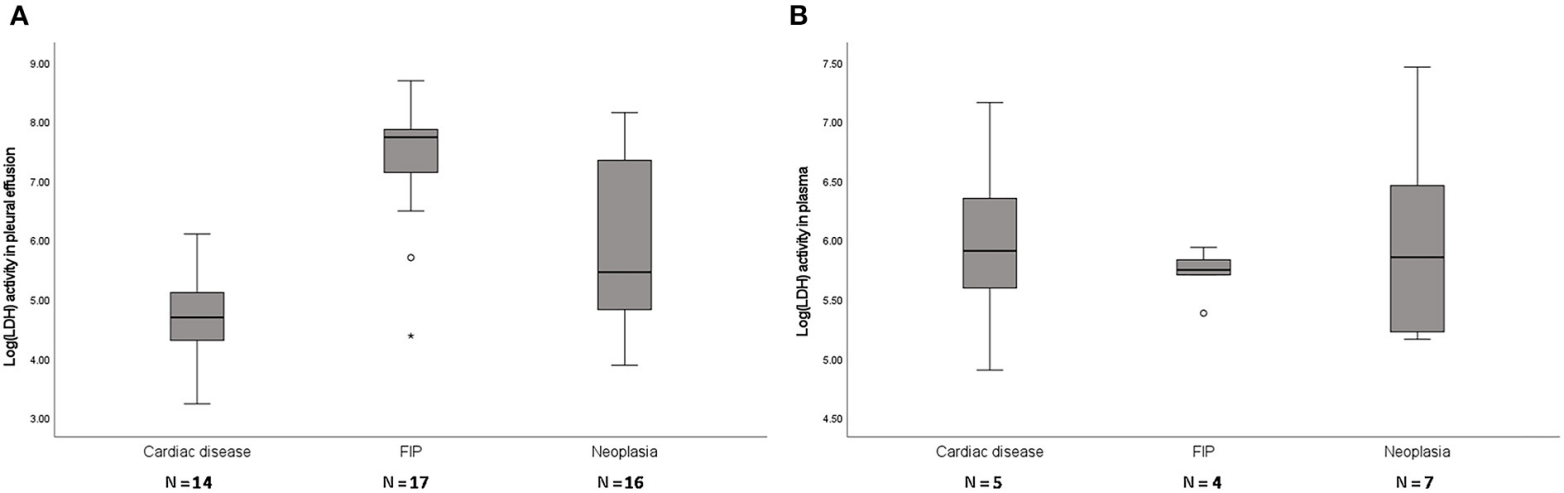
Box plots of LDH levels in pleural fluid **(A)** and plasma **(B)** of cats with modified transudates showing significant differences of LDH activity between groups in pleural fluid (*P* < 0.001) but not in plasma (*P* = 0.766). FIP, feline infectious peritonitis. The boxes represent the 25^th^ and 75^th^ quartiles with a horizontal line at the median. The whiskers represent the range of the data. Open dots represent the outliners. Numbers of analyzed specimens are indicated.

Forty-seven cats with pleural effusion classified as modified transudate were analyzed (Table 1). Cats with FIP had significantly higher pleural fluid LDH (cardiac disease, *P* < 0.0001; neoplasia, *P* = 0.003) and cats with cardiac disease had significantly lower pleural fluid LDH (FIP, *P* < 0.0001; neoplasia, *P* = 0.007). There was no difference in plasma LDH between groups (*P* = 0.766) (Figure 3).

### Receiver operating characteristic curve (ROC) analysis

Receiver operating characteristic analysis was performed in cats with modified transudate pleural effusion for determination of LDH values as predictors of underlying etiology. Pleural fluid LDH in modified transudates distinctly categorized cardiac from non-cardiac diseases (area under the curve (AUC) = 0.873) and FIP from non-FIP diseases (AUC = 0.849). The cut-off values of pleural fluid LDH to distinguish cardiac disease and FIP were <535 and >641 U/L, respectively. Pleural fluid to plasma LDH ratio had less separability for cardiac disease (AUC = 0.857) and FIP (AUC = 0.831). The cut-off value of <0.75 and >1.95 of pleural fluid to plasma LDH ratio could specifically identify cardiac disease and FIP, respectively (Table 2).

**Table 2:** The ROC analysis for determination of LDH values as predictors of etiology in modified transudate pleural effusion.

	**fLDHm**		**fLDH/pLDH**	
	**Cardiac disease**	**FIP**	**Cardiac disease**	**FIP**
AUC	0.878	0.849	0.857	0.831
AUC CI	0.781–0.974	0.730–0.968	0.679–1.000	0.627–1.000
*P*-value	<0.001	<0.001	<0.001	0.034
Cut-off value	<535 U/L	>641 U/L	<0.75	>1.9528
Sens (%)	100	88.24	100	80.00
Sens CI	76.84–100.0	63.56–98.54	59.04–100.00	28.36–99.49
Spec (%)	66.67	80.00	72.73	84.62
Spec CI	48.17–82.04	61.43–92.29	39.03–93.98	54.55–98.08

AUC, area under the curve; CI, 95% confident interval; Sens, sensitivity; Spec, specificity; fLDHm, pleural fluid lactate dehydrogenase activity in modified transudate; fLDH/pLDH, pleural fluid to plasma LDH ratio; FIP, feline infectious peritonitis.

## Discussion

This study investigated and classified 122 pleural fluid samples collected from cats that were clinically diagnosed with different diseases. In our study, neoplasia was the major cause of pleural effusion, which is interestingly in contrast to the recent studies conducted in Europe, reporting cardiac disease as the most common cause (10, 21). The higher number of neoplastic cases with mediastinal lymphoma overrepresented in our study is likely due to the high prevalence of FeLV-associated lymphoma in Thailand and Southeast Asia (22, 23). The role in tumorigenesis of retroviruses has been widely studied in many species and FeLV is one of the most pathogenic oncovirus causing hematopoietic malignancy. The *c-myc* gene, a cellular proto-oncogene, is frequently disrupted by the FeLV genome insertion resulting in oncogene overexpression and uncontrolled proliferation of the affected cells (24). The relative risk of developing lymphoma in FeLV infected cats is approximately 60-fold higher than FeLV-negative cats (25). Mediastinal, ocular, and spinal lymphomas have been reported as commonly affected anatomical locations. In our study, 64% of the cats diagnosed with mediastinal lymphoma were FeLV positive by using a commercial test kit (immunochromatography assay), which detects the p27 antigen of the virus. The most recent study using nested recombinase polymerase amplification (nRPA) to detect exogenous FeLV DNA provirus and RT-PCR to detect FeLV RNA, showed that the FeLV detection rate in the regression phase was increased up to 45.8% compared to the rapid immunochromatographic assay (26). The number of FeLV-positive lymphomatous effusion in our study might have been higher if the molecular detection of the virus was used.

Mammary gland tumors are relatively common neoplasm in female cats and more than 80% of the tumor is extremely malignant with evidence of metastasis to lung and regional lymph nodes (27). In this study, with an additional clinical history of mammary adenocarcinoma in the patients, we were able to diagnose the effusions containing neoplastic epithelial cells as metastatic mammary gland tumors. However, giving a definitive diagnosis of epithelial neoplasms based on cytology is challenging, especially in mesothelial tumor. Mesothelioma is difficult to differentiate from inflammation-induced reactive mesothelial hyperplasia and the final diagnosis should be confirmed with histopathology and immunohistochemistry (28). Other intrathoracic epithelial neoplasms including thymoma and primary lung tumors are very uncommon in cats and are tumors that rarely slough into the pleural effusion.

A positive correlation between plasma and pleural fluid TP concentrations was evident, but this correlation was not observed in LDH. The result indicated that pleural LDH level was not influenced by its level in blood in the same way as TP (29). While the TP concentration is related to its movement between body fluid compartments resulting from altered microvascular permeability, LDH level reflexes disturbances of the cellular integrity induced by pathological conditions. In general, LDH activity in tissues is approximately 500 folds higher than in serum (30). The release of the enzyme from damaged tissues into the extracellular space, particularly into a confined space, can be more relevant to localized injury or inflammation. Lactate dehydrogenase activity is potentially a good marker for local pathologic process and the use of LDH values for diagnostic classification of etiology can be less biased. Measurement of LDH in non-serum samples such as milk and bronchoalveolar lavage fluid has been documented in veterinary medicine and its elevation was associated with cellular injury, inflammation, and malignancy (31, 32). Previous veterinary studies suggested that LDH measured in effusions can be useful to differentiate exudate from transudate as in human medicine (33, 34). However, the LDH level has not been used to identify the underlying condition of the effusion.

It was not surprising that modified transudate was the most common fluid type in our study considering that it is non-specific to the causes and formed by diverse mechanisms. Even though, many veterinary laboratories have routinely classified the effusions into three fluid types, the usefulness of the interpretation of modified transudate is still questionable for veterinary diagnostics. In fact, the cut-off values for body fluid classification are variable between laboratories and are used to evaluate all fluid samples without concerning the species of the animal or the anatomical location of the effusions. In this study, combining LDH measurement with fluid type classification demonstrated a clinically meaningful diagnosis. The cut-off values of LDH level in modified transudates can be practical for identifying cardiac disease and FIP from other etiologies. Considering relatively low cost, but obtaining more informative data from the fluid LDH measurement, this should be added into the routine body fluid analysis and serum biochemistry. We also proposed the cut-off values of pleural fluid to plasma LDH ratio with slightly less separability and higher specificity that can be useful when plasma LDH value is available. Nevertheless, we recommend that to give a diagnosis of modified transudate in pleural effusions, the gross characteristics of the fluid, cytology, and the clinical information should also be accessed together to support the tentative diagnosis.

Some limitations of this study have to be documented. The data in our study were obtained from clinical cases submitted to the Small Animal Hospital, VET CU, Bangkok. The prevalences of underlying diseases and fluid types might not represent all pleural effusion forms in cats in Thailand. Hemolysis in plasma and effusion was omitted from our study considering that hemolysed sample and blood contaminated effusion can cause false increased LDH activity due to release of the enzyme from ruptured erythrocytes (35). It has to be noted that the measurement of LDH in different specimen types may affect the enzyme activity. Plasma is not recommended as a specimen for LDH assay in human medicine. Lactate dehydrogenase activity in plasma is slightly higher than in serum due to leakage of platelet-derived LDH in the plasma (36). However, such evaluation has not been validated in veterinary medicine. Our study used plasma in order to apply the LDH parameter to the routine blood chemistry panel. Regarding the fluid specimen collection, there was no significant difference between the fluid collected in EDTA and plain tube (33). Plasma, serum, and other fluid specimens have been used for LDH assays in veterinary laboratories with their reference ranges. In addition, it needs to be addressed that the proposed LDH cut-off levels in this study are applicable when the pleural fluid is initially classified as a modified transudate. In such circumstance, the LDH level >641 U/L indicates FIP but not pyothorax because the pleural fluid caused by pyothorax is classified as exudate. Adapting the cut-off values for veterinary use should be done under the consideration that our proposed cut-off values might not be applicable when LDH activity is measured in other conditions not indicated in this study.

In summary, findings from this study support the use of LDH values in cats as an adjunct test to the routine fluid analysis. We have shown that pleural fluid LDH is not affected by plasma LDH and can be a useful marker for differentiating the causes of modified transudate pleural effusions in cats. We propose that the pleural fluid LDH level of <535 and >641 U/L can be used to identify cardiac disease and FIP modified transudate effusions, respectively. Thoracocentesis is a minimally invasive procedure that is not only used for therapeutic, but also diagnostic purpose. Veterinarians should fully utilize laboratory data from fluid analysis when the specimen is available. Supporting information of clinical signalments and comprehensive fluid analysis are highly beneficial for veterinarians in narrowing down the list of differential diagnoses of pleural effusion.

## Data availability statement

The original contributions presented in the study are included in the article/Supplementary material, further inquiries can be directed to the corresponding author/s.

## Ethics statement

The study was approved by Chulalongkorn University Animal Care and Use Committee (Approval No. 2131016) in accordance with the faculty regulations. Written informed consent was obtained from the owners for the participation of their animals in this study.

## Author contributions

HM conducted data acquisition, contributed to data analysis, and drafted the manuscript. VH contributed to data analysis and provided statistical guidance during the drafting of the manuscript. JS assisted in data acquisition. ARu advised on data analysis and provided scientific guidance. ARa established the concept of the project, contributed to data analysis, and drafted the manuscript. All authors contributed to the article and approved the submitted version.

## Funding

HM received master's degree support from the Chulalongkorn University Graduate Scholarship Program for ASEAN and Non-ASEAN Countries and Chulalongkorn University Graduate School Thesis Grant (3/2021). This study was supported by Veterinary Science Research Fund, Chulalongkorn University (RG2/2563).

## Conflict of interest

The authors declare that the research was conducted in the absence of any commercial or financial relationships that could be construed as a potential conflict of interest.

## Publisher's note

All claims expressed in this article are solely those of the authors and do not necessarily represent those of their affiliated organizations, or those of the publisher, the editors and the reviewers. Any product that may be evaluated in this article, or claim that may be made by its manufacturer, is not guaranteed or endorsed by the publisher.
