# Supplementary material for: Diagnostic utility of LDH measurement for determining the etiology of modified transudate pleural effusion in cats
Source: Front Vet Sci. 2022 Nov 3;9:1044192. doi: 10.3389/fvets.2022.1044192 (PMC9669425; doi:10.3389/fvets.2022.1044192)
Supplement: Supplementary file 1 [file Table_1.DOCX]

**Supplementary material**

Comparison between parameters in pleural fluid and plasma and definitive etiology of 108 cats with pleural effusions

| Parameters | | Etiologies | | | | |
| --- | --- | --- | --- | --- | --- | --- |
|  |  | Neoplasia  (a) | CD  (b) | FIP  (c) | Pyothorax (d) | Comparison between groups  (*P*-value) |
| Age (Year) | | n = 47  3  0.58–16 | n = 24  5  (1–14) | n = 22  1.25  (0.33–13) | n = 15  1  (0.33–7) | b-c (<0.0001) b-d (0.001) |
| Dead  (Number of cats) | | n = 47  8  (17.02%) | n = 24  8  (33.33%) | n = 22  5  (22.73%) | n = 15  3  (20%) | – |
| Pleural fluid | TNCC (cells/µl) | n = 46  14800  (75–20000) | n = 24  675  (50–4900) | n = 21  2925  (225–18600) | n = 15  20000  (19925–20000) | b-a (<0.0001)  b-d (<0.0001)  c-a (0.031)  c-d (<0.0001) |
|  | TP (g/dl) | n = 47  4.10  (1.50–12.60) | n = 24  2.30  (0.80–5.00) | n = 22  6.35  (3.20–10.80) | n = 15  4.90  (0.60–10.60) | b-a (<0.0001)  b-d (0.001)  b-c (<0.0001)  a-c (0.004) |
|  | LDH (U/L) | n = 47  888.50  (48.30–8141.20) | n = 24  92.75  (25.20–443.70) | n = 22  2358.20  (79.40–6587.00) | n = 15  5292.10  (75.30–34948.80) | b-a (<0.0001)  b-c (<0.0001)  b-d (<0.0001)  a-d (0.035) |
| Plasma | TP (g/dl) | n = 20  5.90  (4.10–7.10) | n = 11  5.80  (4.70–7.40) | n = 12  7.25  (4.70–8.70) | n = 9  6.20  (4.20–7.90) | b-c (0.050)  a-c (0.041) |
|  | LDH (U/L) | n = 17  404.60  (173.90–1736.70) | n = 9  376.50  (134.00–1286.30) | n = 7  340.50  (216.80–1463.10) | n = 5  445.90  (264.60–2646.60) | – |

Data are median (range). The comparison between variables and etiologies was performed using Kruskal–Wallis test and post-hoc analysis using Mann–Whitney U test. Only the significant comparisons (*P* <0.05) are described. CD, cardiac disease; FIP, feline infectious peritonitis; TNCC, total nucleated cell count; TP, total protein concentration; LDH, lactate dehydrogenase level.
